# LRRK2^G2019S^ Gene Mutation Causes Skeletal Muscle Impairment in Animal Model of Parkinson's Disease

**DOI:** 10.1002/jcsm.13604

**Published:** 2024-09-23

**Authors:** Yiying Hu, Huijia Yang, Chunli Song, Lulu Tian, Panpan Wang, Tianbai Li, Cheng Cheng, Murad AlNusaif, Song Li, Zhanhua Liang, Weidong Le

**Affiliations:** ^1^ Key Laboratory of Liaoning Province for Research on the Pathogenic Mechanisms of Neurological Diseases The First Affiliated Hospital of Dalian Medical University Dalian China; ^2^ Department of Neurology The First Affiliated Hospital of Dalian Medical University Dalian China

**Keywords:** electromyography, LRRK2^G2019S^ mutation, mitochondrial impairment, Parkinson's disease, skeletal muscle impairment

## Abstract

**Background:**

While the gradually aggravated motor and non‐motor disorders of Parkinson's disease (PD) lead to progressive disability and frequent falling, skeletal muscle impairment may contribute to this condition. The leucine‐rich repeat kinase2 (LRRK2) is a common disease‐causing gene in PD. Little is known about its role in skeletal muscle impairment and its underlying mechanisms.

**Methods:**

To investigate whether the mutation in LRRK2 causes skeletal muscle impairment, we used 3‐month‐old (3mo) and 14‐month‐old (14mo) LRRK2^G2019S^ transgenic (TG) mice as a model of PD, compared with the age‐matched littermate wild‐type (WT) controls. We measured the muscle mass and strength, ultrastructure, inflammatory infiltration, mitochondrial morphology and dynamics dysfunction through behavioural analysis, electromyography (EMG), immunostaining, transmission electron microscopy (TEM) and other molecular biology techniques.

**Results:**

The 3mo‐TG mice display mild skeletal muscle impairment with spontaneous potentials in EMG (increased by 130%, *p* < 0.05), myofibre necrosis (*p* < 0.05) and myosin heavy chain‐II changes (reduced by 19%, *p* < 0.01). The inflammatory cells and macrophage infiltration are significantly increased (CD8a^+^ and CD68^+^ cells up 1060% and 579%, respectively, both *p* < 0.0001) compared with the WT mice. All of the above pathogenic processes are aggravated by aging. The 14mo‐TG mice EMG examinations show a reduced duration (by 31%, *p* < 0.01) and increased polyphasic waves of motor unit action potentials (by 28%, *p* < 0.05). The 14mo‐TG mice present motor behavioural deficits (*p* < 0.05), muscle strength and mass reduction by 37% and 8% (*p* < 0.05 and *p* < 0.01, respectively). A remarkable increase in inflammatory infiltration is accompanied by pro‐inflammatory cytokines in the skeletal muscles. TEM analysis shows muscle fibre regeneration with the reduced length of sarcomeres (by 6%;*p* < 0.05). The muscle regeneration is activated as Pax7^+^ cells increased by 106% (*p* < 0.0001), andmyoblast determination protein elevated by 71% (*p* < 0.01). We also document the morphological changes and dynamics dysfunction of mitochondria with the increase of mitofusin1 by 43% (*p* < 0.05) and voltage‐dependent anion channel 1 by 115% (*p* < 0.001) in the skeletal muscles of 14mo‐TG mice.

**Conclusions:**

Taken together, these findings may provide new insights into the clinical and pathogenic involvement of LRRK2^G2019^ mutation in muscles, suggesting that the diseases may affect not only midbrain dopaminergic neurons, but also other tissues, and it may help overall clinical management of this devastating disease.

## 1 Introduction

Parkinson's disease (PD) is the second most common neurodegenerative disease, pathologically characterized by progressive dopaminergic (DAergic) neuron loss and α‐synuclein aggregations in the substantia nigra [1]. The clinical features of PD are not only cardinal motor symptoms (bradykinesia, rigidity, rest tremor and postural instability) but also various non‐motor features [2]. During the disease progression, fatigue [3], muscular pain [4], sarcopenia [5], dynapenia [6] and muscle wasting [7] are common, which increase the risk of fall incidence [8], fractures [9] and disability. In the late stage, falls and dysphagia are significant negative factors predicting a poor outcome [10]. However, there are no universal diagnostic criteria for muscle wasting or myopathy in PD around the world, and it is easy to make a misdiagnosis by clinicians.

Currently, there is little known about muscle impairment in PD. Several case reports about camptocormia and dropped head syndrome, a rare type of dystonia with paraspinal muscle weakness, imply that focal myositis in paraspinal muscles may occasionally be present in PD [S1‐S2, 11]. There is a report showing the myopathic changes in the trapezius, erector spinal muscles and other unaffected muscles under the electromyography (EMG) examination [S3]. In a study of PD patients with camptocormia, paraspinal muscles show inflammatory infiltrations and mitochondrial abnormalities, including ragged‐red fibres confirmed by biopsy [12, S4]. Similarly, dysphagia is another frequent clinical symptom experienced in 53%–80% of PD patients during the disease course [13]. It may cause serious complications such as sialorrhea, silent aspirators, malnutrition and aspiration pneumonia [14]. Other reports of pharyngeal muscle atrophy [15] and inspiratory muscle weakness in PD may cause chronic hypoxemia and pulmonary dysfunction [S5]. A few lower‐limb skeletal muscle biopsies from PD patients reveal the exaggerated type I myofibre grouping with the dysfunction of the mitochondrial respiratory chain [16, S6, S7]. These studies provide some evidence of muscle impairment in PD, but the pathophysiological mechanisms remain elusive.

The milestone discovery of the α‐synuclein gene (SNCA) highlights the genetic aetiology of PD [17]. Several reports have confirmed that myofibre atrophy [18] or the dysfunction of neuromuscular junctions can be found in a mouse model overexpressing human mutant α‐synuclein [19]. Similarly, a model of DJ‐1‐deficient mouse develops muscle atrophy and muscle strength reduction [20]. However, little is known about other PD‐related genes with muscle impaired disorders.

The leucine‐rich repeat kinase 2 (LRRK2) gene causes the most common familial cases in autosomal dominant PD, and LRRK2 Gly2019Ser (LRRK2^G2019S^) consists of 4% of familial and 1% of sporadic PD worldwide [21]. Aging plays a vital role in the pathogenesis [22]. The LRRK2 gene is a large multidomain protein containing various protein–protein interaction domains, GTPase activity and serine/threonine protein kinase domain. LRRK2^G2019S^ mutation is located in the kinase domain with enhanced kinase activity [S8], which is associated with inflammatory pathway, mitochondrial dysfunction and altered cytoskeletal dynamics [23, S9]. Furthermore, LRRK2 is highly expressed not only in the brain but also in the circulating immune cells, lungs and kidneys [S10, S11]. Besides, LRRK2^G2019S^ is a risk gene of inflammatory bowel disease [24, S12]. However, its precise pathophysiological mechanisms for PD and the role in skeletal muscles are still not fully understood. LRRK2^G2019S^ transgenic (TG) mice at 12 months in our previous study display a moderate DAergic dysfunction [25]. Our present study demonstrates that the LRRK2 ^G2019S^ mice exhibit skeletal muscle impairment with slight inflammatory infiltration at the early stage of disease, and aggregated during aging with weak muscle strength, myofibre necrosis and regeneration, mitochondrial damage and dynamics dysfunction.

## 2 Materials and Methods

### 2.1 Animals

LRRK2 ^G2019S^ mice [B6. Cg‐Tg (Lrrk2*G2019S) 2Yue/J, Stock No:012467] were obtained from Jackson Laboratory (Bar Harbour, United States). The male mice were randomly divided into four groups based on age and genotype: 3‐month‐old TG mice (3mo‐TG), 3‐month‐old WT mice (3mo‐WT), 14‐month‐old TG mice (14mo‐TG) and 14‐month‐old WT mice (14mo‐WT). The number of animals used in this study was given in each method section. The details of the genotype information are described in Data S1.

All mice in our experiments were approved by the Institutional Animal Care Committee of Dalian Medical University.

### 2.2 Behavioural Analysis

All four groups of mice were examined by rotarod test, pole‐climbing test and forelimb grip strength test. The results were obtained from seven to nine mice for each group. Further details are addressed in Data S1.

### 2.3 Electromyography Examination

EMG recordings provide an objective tool for differential localization diagnosis of neuromuscular disease [26]. In our study, we used needle electromyography (nEMG), motor nerve conduction velocity (MCV) and repetitive nerve stimulation (RNS) to examine the electroactivity in gastrocnemius muscles and sciatic nerve for all four groups of mice. There were six to eight mice in each group at the age of 14 months and three mice in each group at 3 months. Further details are described in Data S1.

### 2.4 Histological Examination, Immunohistochemical and Immunofluorescence Staining

For the pathological process, we used haematoxylin and eosin (H&E), modified gomori trichrome (MGT) and immunohistochemical (IHC) staining to investigate the histology, abnormal mitochondria and inflammatory infiltration pathology of muscle fibres in our four groups of mice. The results were obtained from three to six mice for each group. In addition, we used immunofluorescence (IF) staining to examine the type II muscle fibre pathology and regeneration. The details of those examinations are described in Data S1.

### 2.5 Transmission Electron Microscopy Analysis

Two groups of mice (14mo‐TG and 14mo‐WT) in our study were examined using transmission electron microscopy (TEM). There were three mice in each group, and the details of the TEM examination are described in Data S1.

### 2.6 Protein Extraction and Western Blotting

We performed western blotting in the gastrocnemius muscle extracts obtained from four groups of mice to determine the inflammation and mitochondrial function at the molecular levels. The results were obtained from four mice for each group, and the details of the experiments are described in Data S1.

### 2.7 Statistical Analysis

All data were analysed using GraphPad Prism 8.0 software (GraphPad Software Inc, USA), and quantitative data were expressed as means ± SEM. The data from behavioural analyses, EMG and biochemical assay results were analysed by two‐way ANOVA followed by Sidak's multiple comparisons tests. Student's *t*‐tests were used to analyse the data from TEM. *p* < 0.05 was considered as significant. All experiments were repeated at least three times. Our sample sizes were similar to those of the publications previously.

## 3 Results

### 3.1 LRRK2^G2019S^ Mice Develop an Age‐Dependent Decline in the Motor Performance and Skeletal Muscle Strength

**FIGURE 1:**
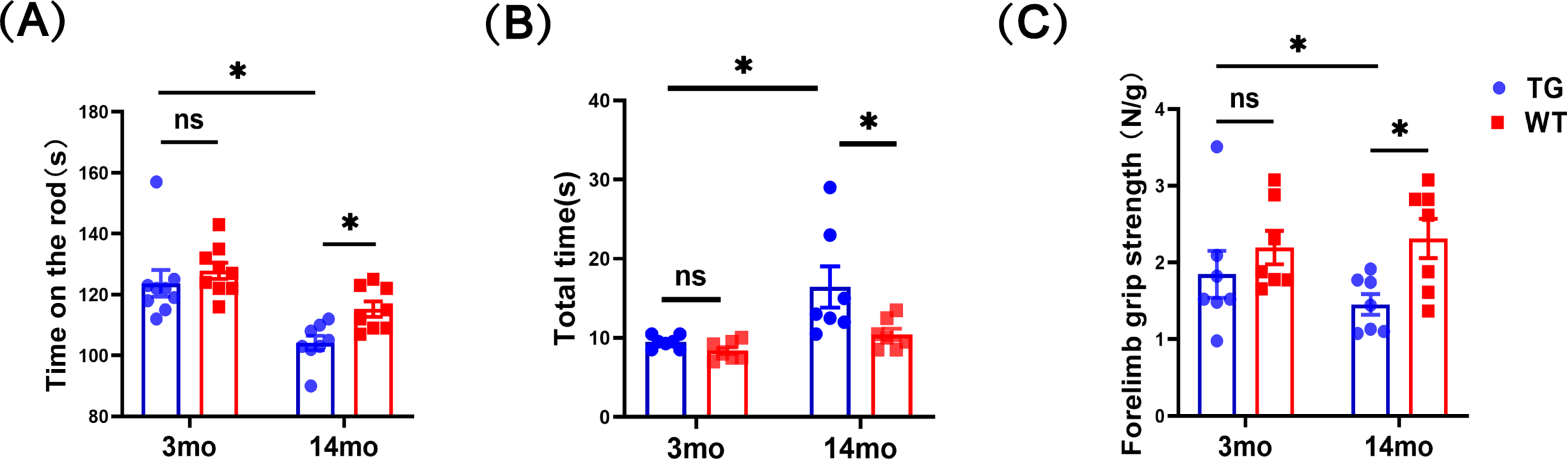
Motor performance in LRRK2^G2019S^ mice. (A) The latency to fall from rotarod. (B) The total time including turn around and climbing down from the top to the ground. (C) Forelimb grip strength standardization by body weight. *n* = 7–9 mice per group. Data were analysed by two‐way ANOVA and presented as the mean ± SEM. ns, non‐significant, *p < 0.05, **p < 0.01.

The rotarod test was first used to assess the motor function of LRRK2^G2019S^ mice. We found that the 3mo‐TG and 3mo‐WT mice performed equally well, but the 14mo‐TG mice showed significantly less time compared with the 14mo‐WT mice on the road (decreased by 10%, *p* < 0.05; Figure 1A). Besides, we applied the pole‐climbing test to evaluate the motor balance, which was dominated by the strength of the forelimbs. As a result, only the 14mo‐TG mice required a longer total time spent on the pole compared with the age‐matched WT mice (increased by 37%, *p* < 0.05; Figure 1B), while the 3mo‐TG and 3mo‐WT mice displayed similar time. Consistently, we measured the strength of forelimb grip and found no significant difference between the 3mo‐TG and 3mo‐WT mice. However, the mean strength was markedly decreased in the 14mo‐TG mice by 37% compared with the 14mo‐WT mice (*p* < 0.05; Figure 1C). Together, our data reveal that LRRK2^G2019S^ mice exhibite an age‐dependent decline in motor balance and skeletal muscle strength.

### 3.2 EMG Examination Shows Age‐Dependent Myogenic‐Like Changes in the LRRK2^G2019S^ Mice

**FIGURE 2:**
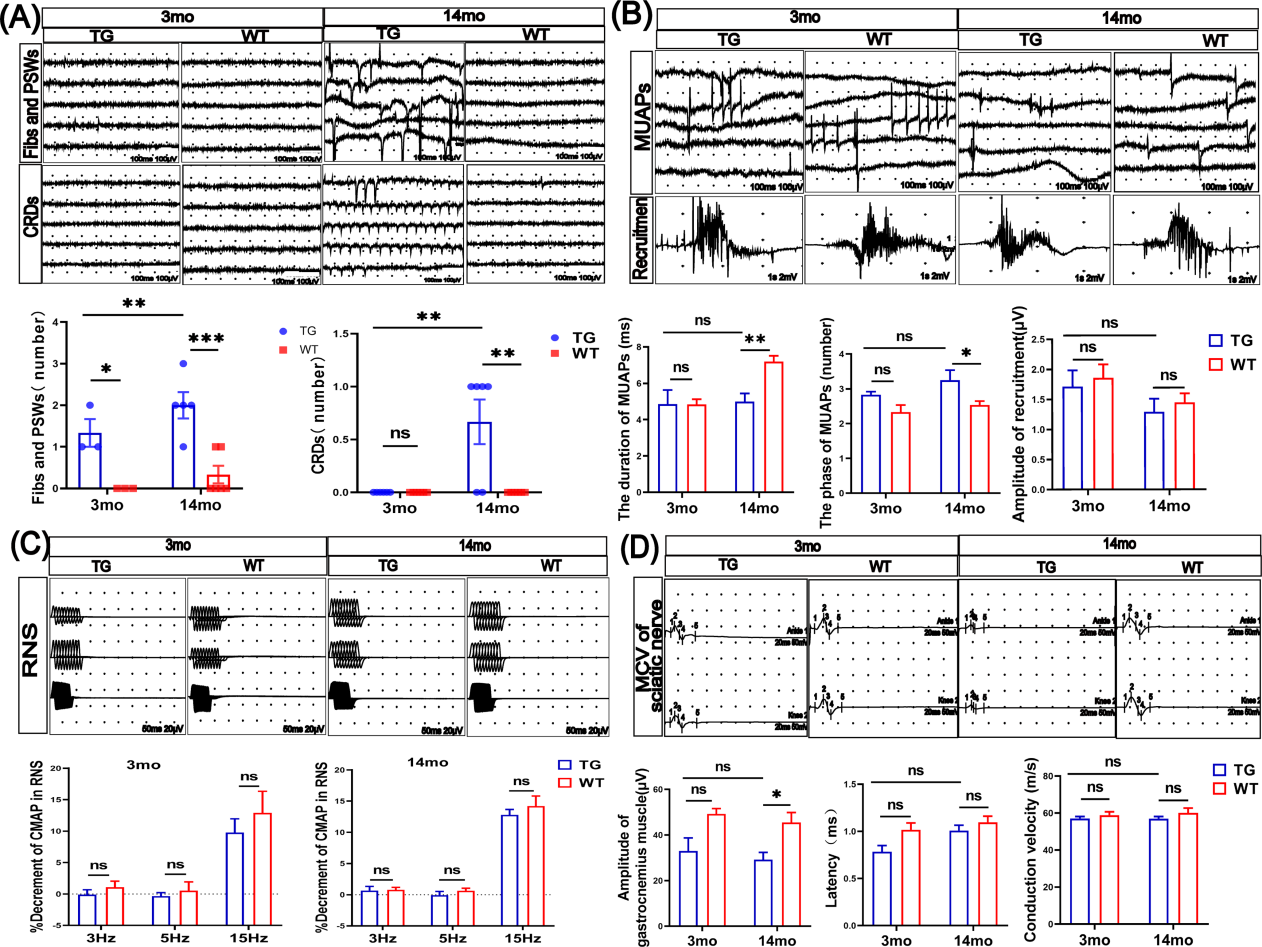
The EMG tracing with the age‐dependent myogenic‐like changes in the gastrocnemius muscles of LRRK2^G2019S^ mice. (A) The representative images of spontaneous potential discharges include fibrillation potentials (Fibs), positive sharp waves (PSWs), and CRDs. Quantitative analysis of the number of spontaneous potential discharges. (B) The representative images of MUAPs and recruitment. Quantitative analysis of the mean duration, the mean phase of MUAPs, and the amplitude of recruitment. (C) The representative images of RNS. Quantitative analysis of RNS at 3, 5, and 15 Hz of 3‐ and 14‐month‐old mice. (D) The representative images of motor conduction velocity of sciatic nerves. Quantitative analysis of the amplitude, latency and conduction velocity. 3‐month‐old, *n* = 3 mice per group; 14‐month‐old, *n* = 6–8 mice per group. Data were analysed by two‐way ANOVA and presented as the mean ± SEM. ns, non‐significant, *p < 0.05, **p < 0.01.

Because LRRK2^G2019S^ mice showed skeletal muscle dysfunction, we attempted to detect muscle electroactivity by EMG. Specifically, we found that the number of spontaneous potential discharges (Figure 2A) was significantly increased in all gastrocnemius muscles in the 3mo‐TG mice (by 130%, *p* < 0.05) and the 14mo‐TG mice (by 560%, *p* < 0.01). Moreover, the complex repetitive discharges (CRDs, the marker of chronic muscle impairment) were only detected in the 14mo‐TG mice (increased by 50%, *p* < 0.01; Figure 2A). Furthermore, the mean duration of motor unit action potentials (MUAPs) was decreased by 31% (*p* < 0.01; Figure 2B), polyphasic waves were increased by 28% (*p* < 0.05; Figure 2B) in the 14mo‐TG mice compared with 14mo‐WT mice, but there were no significant changes in the 3‐month‐old mice (Figure 2B). Also, we found no significant differences in 3mo‐TG and 14mo‐TG mice with the amplitude of recruitment compared with the age‐matched controls (Figure 2B). These results demonstrate that LRRK2^G2019S^ mice may have hyperexcitable muscle membranes [26] at a young age, which are aggravated with the diminished number of electrically responsive muscle fibres during aging.

In order to eliminate the abnormal formation of MUAPs caused by the dysfunction of neuromuscular junctions, RNS was performed on all four groups of mice (Figure 2C). As expected, there was no significant difference in low‐frequency and high‐frequency stimulation between the TG and WT mice (Figure 2C). Next, we further analysed the MCV of sciatic nerves (Figure 2D), an essential component of the integrity of neuromuscular diseases. At 3 months old, the amplitude of CMAPs of gastrocnemius muscles in TG and WT mice was equal but reduced by 33% in the TG mice at 14 months old (*p* < 0.05; Figure 2D). Besides, there were no significant changes in latency or conduction velocity (Figure 2D) both in the 3mo‐TG and 14mo‐TG mice compared with the age‐matched controls, indicating that the myelin function of sciatic nerves and the neuromuscular junction were integrated.

### 3.3 LRRK2^G2019S^ Mice Show an Age‐Dependent Gastrocnemius Mass Reduction and Myofibre Necrosis

**FIGURE 3:**
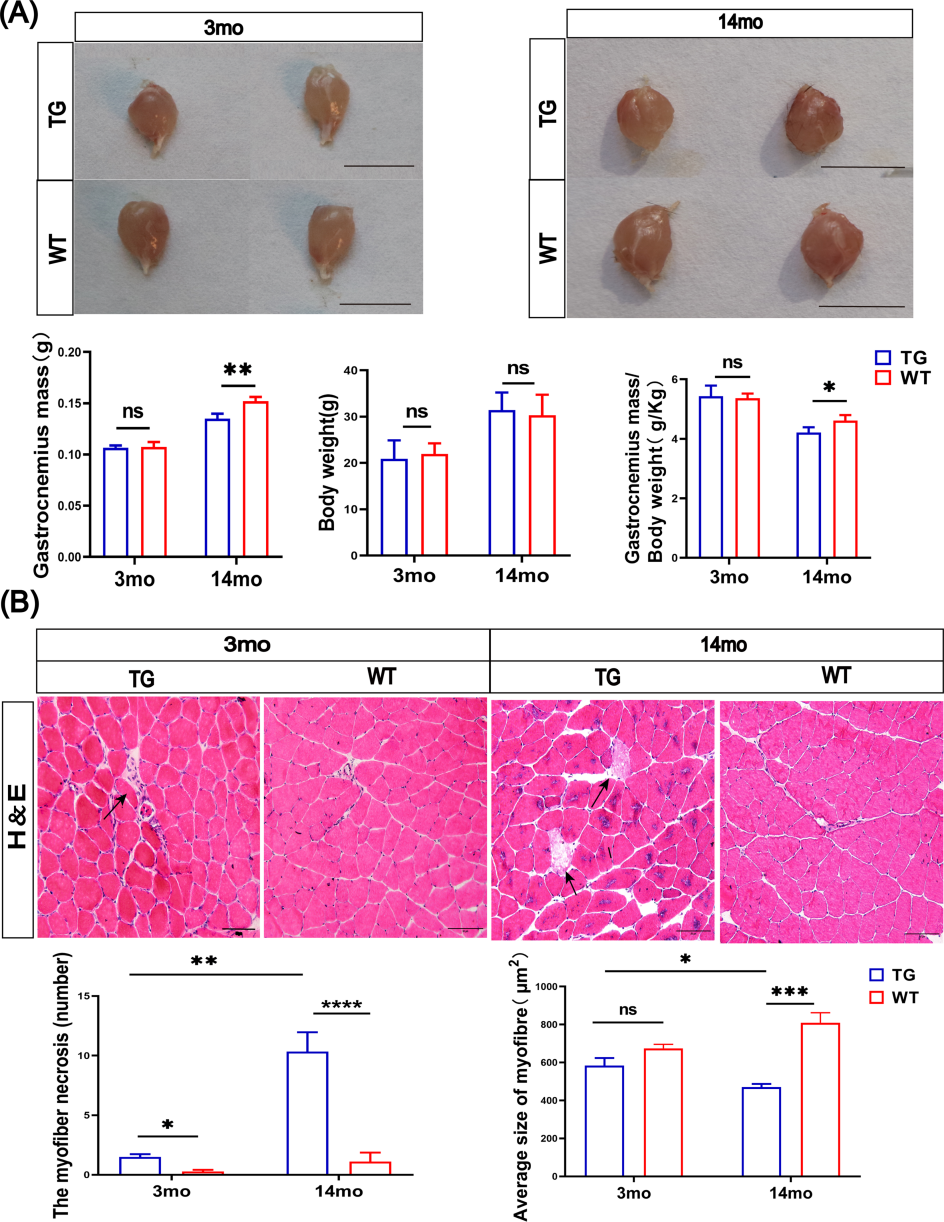
LRRK2^G2019S^ mice display age‐dependent muscle mass reduction and myofibre necrosis. (A) Morphology of gastrocnemius muscles of TG and WT mice at 3 and 14 months. Scale bars 1 cm. Quantitative analysis of the gastrocnemius muscle mass, body weight and gastrocnemius muscle mass/body weight (g/kg). *n* = 6 gastrocnemius muscles from 3 mice per group. (B) Representative images of H&E staining of biceps femoris sections. H&E staining showed the necrosis of the myofibres (black arrows). *n* = 6 slices per mouse and 3 mice per group; scale bars 50 μm. Quantification of the myofibres with inflammatory infiltration, average myofibre size. Data were analysed by two‐way ANOVA and presented as the mean ± SEM. ns, non‐significant, *p < 0.05, **p < 0.01, ***p < 0.001.

Moreover, our results showed that the gastrocnemius muscle mass was significantly decreased by 13% in the 14mo‐TG mice (*p* < 0.01; Figure 3A), and the mass was also reduced by 8% after body weight standardization (*p* < 0.05; Figure 3A). However, there was no significant difference regarding the gastrocnemius muscle mass and body weight between 3mo‐TG and 3mo‐WT mice (Figure 3A).

Next, we conducted H&E staining, showing a clear myofibre necrosis in the 3mo‐TG mice (*p* < 0.05; Figure 3B), and severe necrosis and inflammatory infiltration increased by 827% in the 14mo‐TG mice (*p* < 0.0001; Figure 3B) compared with the age‐matched WT mice. Additionally, the average area of myofibre was decreased by 39% (*p* < 0.001; Figure 3B) in 14mo‐TG mice versus 14mo‐WT mice, and not in 3‐month‐old mice. These findings suggest age‐dependent alteration of chronic inflammatory response in the TG mice.

### 3.4 Age‐Dependent Alteration of Skeletal Muscle Structure in the LRRK2^G2019S^ Mice

**FIGURE 4:**
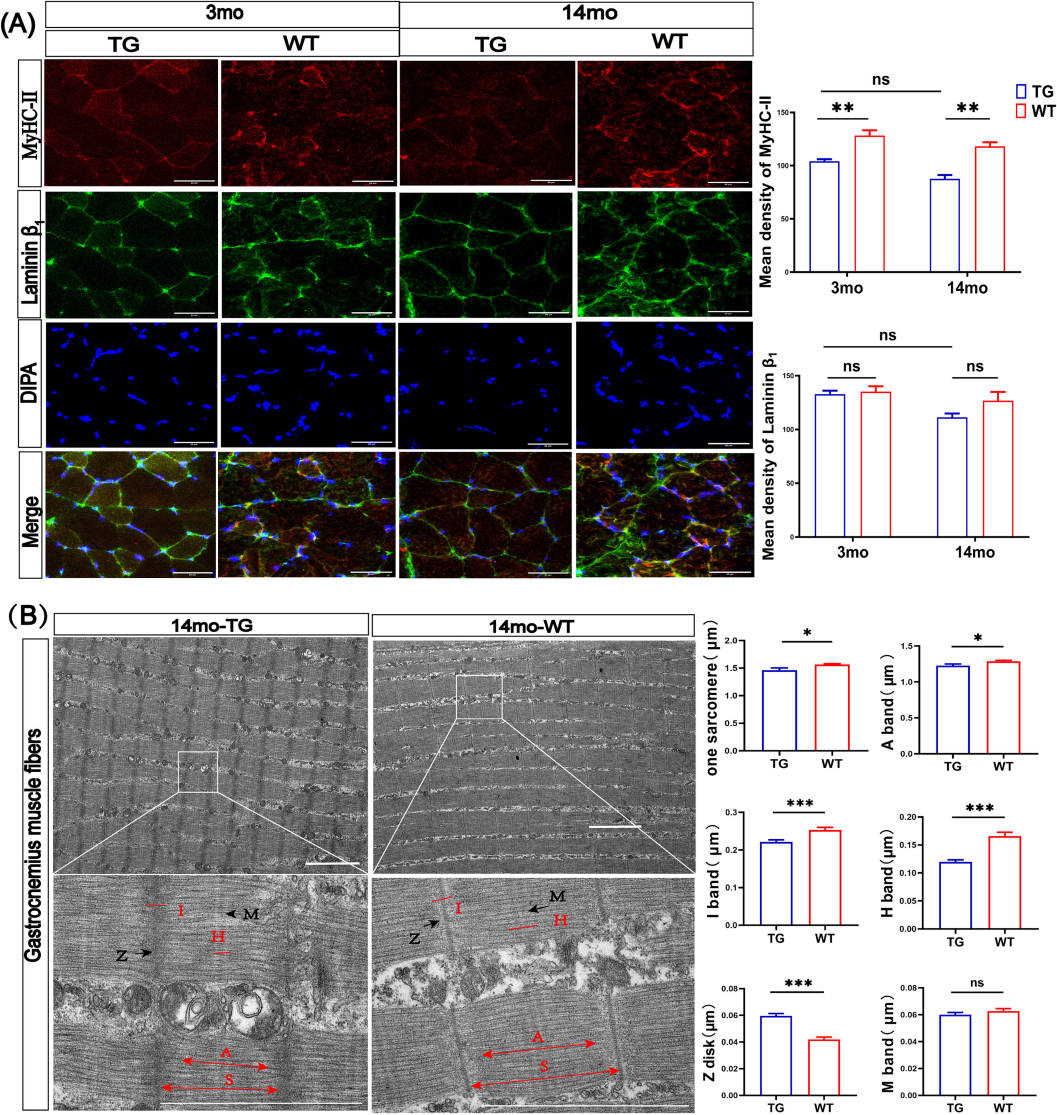
Structure changes in the skeletal muscles of LRRK2^G2019S^ mice during aging. (A) Immunofluorescence analysis for MyHC‐II expression in biceps femoris muscles (Red) together with Laminin β_1_ (Green). The nucleus was labelled with DAPI (Blue). *n* = 7–14 slices per mouse and 3 mice per group, scale bars 50 μm. Quantification of the mean integrated density of MyHC‐II, Laminin β1. (B) TEM images of the gastrocnemius muscles, double arrow: sarcomere (S), A band (A); red line: I band (I), H zone (H); black arrow: Z disk (Z) and M line (M). Quantification of the sarcomere, A band, I band, H zone, Z disk and M line. Scale bar 2 μm. *n* = 10–12 slices per mouse and 3 mice per group. IF data were analysed by two‐way ANOVA; TEM data were analysed using Student's *t*‐test. All data were presented as the mean ± SEM. ns, non‐significant, *p < 0.05, **p < 0.01, ***p < 0.001.

To analyse the skeletal muscle structure, we determined the expression level of myosin heavy chain‐II (MyHC‐II), which was the composition of fast skeletal muscles, and measured its contractile function (Figure 4A). We found a 19% and 26% reduction in the 3mo‐TG and 14mo‐TG mice compared with age‐matched WT mice, respectively (both *p* < 0.01; Figure 4A). Meanwhile, the level of the laminin β_1_ chain, the components of the basal layer of skeletal muscle cells, was not significantly changed (Figure 4A). Besides, we further analysed the ultrastructure of gastrocnemius muscles with TEM (Figure 4B), showing a decreased of sarcomeres length by 6% (*p* < 0.05), I band width by 12% (*p* < 0.001), A band width by 5% (*p* < 0.05) and H zone width by 27% (*p* < 0.001) in the 14mo‐TG mice compared with the age‐matched WT mice (Figure 4B). The Z disk spread slightly by 44% (*p* < 0.001; Figure 4B), and the M bands were generally integrated (*p* > 0.05; Figure 4B). These structural abnormalities in the LRRK2^G2019S^ mice indicate progressive deterioration in skeletal muscle impairment.

### 3.5 Age‐Dependent Myofibre Abnormality in the LRRK2^G2019S^ Mice Accompanied by Inflammatory Infiltration

**FIGURE 5:**
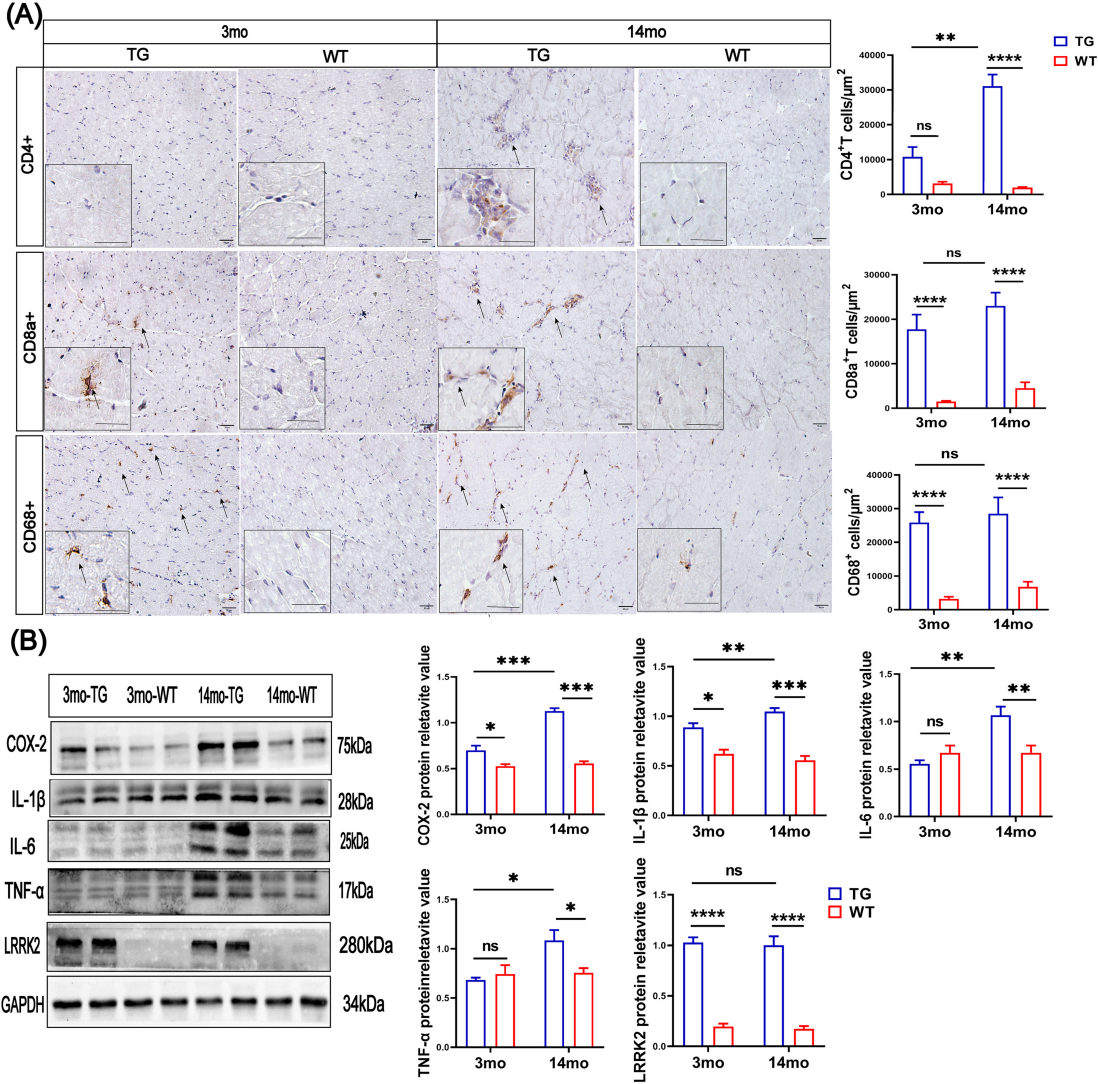
Macrophages, lymphocyte infiltration and pro‐inflammatory cytokines levels in the biceps femoris muscles of LRRK2^G2019S^ mice. (A) Immunohistochemistry of CD4^+^T‐cell, CD8a^+^T‐cell and CD68^+^‐cell expression in biceps femoris muscles, scale bars 50 μm. *n* = 10–12 slices per mouse and 3 mice per group. Quantification of CD4^+^T‐cell, CD8a^+^T‐cell and CD68^+^‐cell. (B) Western blot detecting pro‐inflammatory cytokines of COX‐2, IL‐1β, IL‐6, TNF‐α and LRRK2 protein expressions in gastrocnemius muscles. *n* = 4 mice per group. Experiments were repeated three times. Quantification of IL‐1β, IL‐6, TNF‐α, COX‐2 and LRRK2 protein expressions relative to GAPDH. Data were analysed by two‐way ANOVA and presented as the mean ± SEM. ns, non‐significant, *p < 0.05, **p < 0.01, ***p < 0.001.

We then further performed IHC staining on the biceps femoris muscles to detect the inflammatory infiltration with classical markers CD68 of macrophages, CD4 and CD8a as the classical lymphocyte markers (Figure 5A). Our data demonstrated that CD4‐positive cells were markedly increased by 140% in 14mo‐TG mice compared versus 14mo‐WT mice (*p* < 0.0001; Figure 5A), but not increased in the 3mo‐TG mice. CD8a positive cells were increased by 1060% and 409% in 3mo‐TG and 14mo‐TG mice, respectively (both *p* < 0.0001; Figure 5A). Similarly, CD68 positive cells infiltrated myofibres were increased by 579% and 785% in the endomysial and perimysial regions of 3mo‐TG and 14mo‐TG mice, respectively (both *p* < 0.0001; Figure 5A).

In addition, we measured the pro‐inflammatory cytokines, such as tumour necrosis factor‐α (TNF‐α), interleukin‐1β (IL‐1β), interleukin‐6 (IL‐6) and inflammatory cytokines cyclooxygenase‐2 (COX‐2) as the markers of chronic inflammation (Figure 5B). There was a significant increase of COX‐2 by 15% in the 3mo‐TG mice (*p* < 0.05) and by 104% in the 14mo‐TG mice, respectively (*p* < 0.001; Figure 5B); there was also a significant increase of IL‐1β level by 33% in the 3mo‐TG mice (*p* < 0.05; Figure 5B) and by 90% in the 14mo‐TG mice, respectively (*p* < 0.001; Figure 5B). Besides, an increased level of TNF‐α (by 44%, *p* < 0.05) and IL‐6 (by 58%, *p* < 0.01) was found in the 14mo‐TG mice, but not in the 3mo‐TG mice (Figure 5B). These biomarkers are essential for determining the development of chronic inflammation and skeletal myofibre necrosis.

### 3.6 Regeneration Process and Reactive Satellite Cells in the Skeletal Muscles of LRRK2^G2019S^ Mice

**FIGURE 6:**
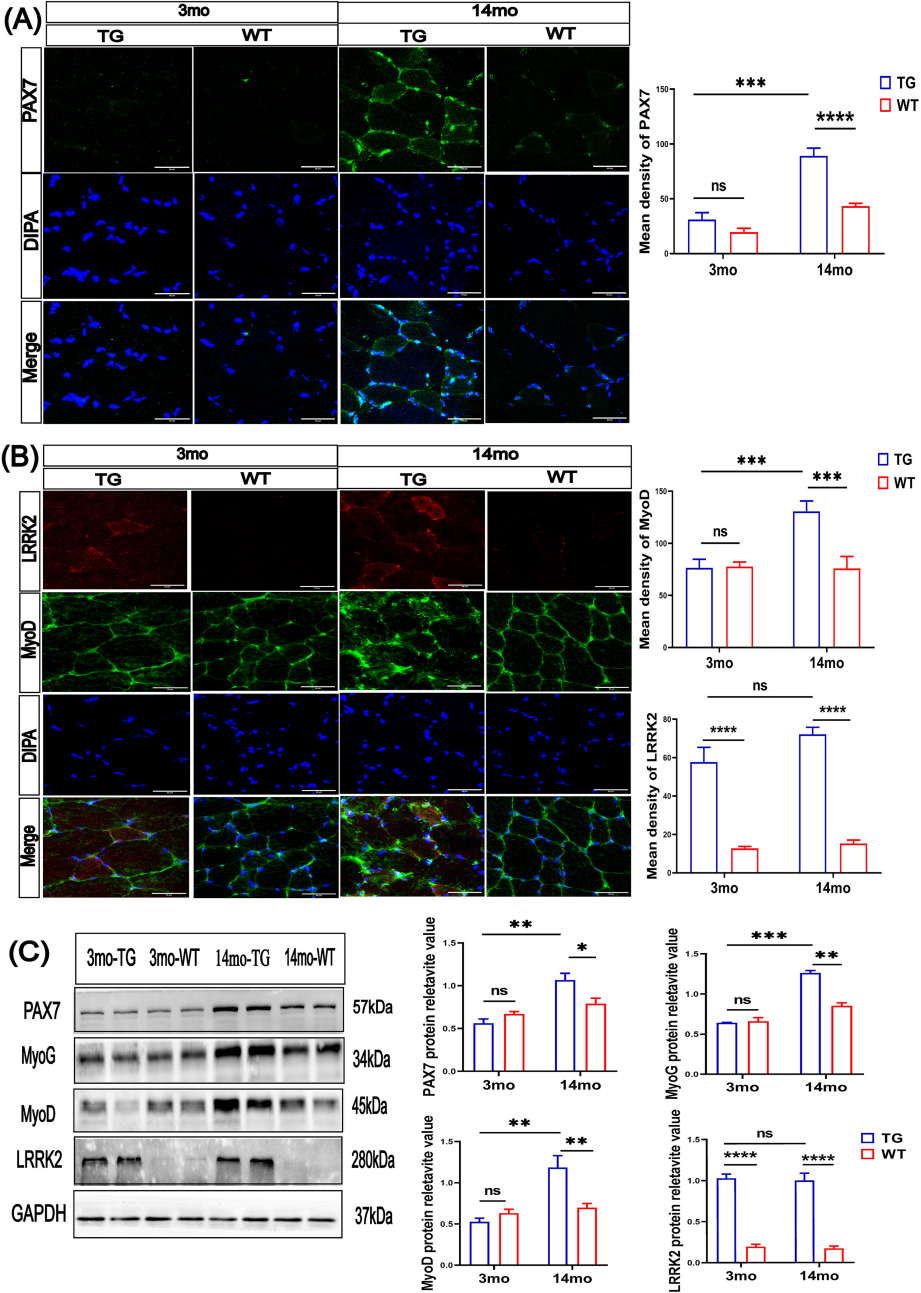
Activated satellite cells and muscle regeneration in the biceps femoris muscles of LRRK2^G2019S^ mice. (A) Immunofluorescence of PAX7^+^cell in biceps femoris muscles (green), the nuclei were labelled with DAPI (Blue). Quantification of mean integrated density of PAX7. (B) Immunofluorescence of MyoD (green) and LRRK2 (red), nucleus was labelled with DAPI (Blue). Quantification of the integrated density of MyoD, and LRRK2. Scale bar 50 μm. *n* = 10–14 slices per mouse and 3 mice per group. (C) Western blot analysis for PAX7, MyoG, MyoD, LRRK2 and GAPDH in gastrocnemius muscles, *n* = 4 mice per group. Experiments were repeated three times. Quantification of PAX7, MyoG, MyoD and LRRK2 protein expressions relative to GAPDH. Data were analysed by two‐way ANOVA and presented as the mean ± SEM. ns, non‐significant, *p < 0.05, **p < 0.01, ***p < 0.001.

Skeletal muscles have a potent ability to repair injuries, following the injury‐repair‐regeneration strictly. Satellite cells are first activated and proceed to the proliferative stage. Muscle‐specific transcription factors regulate the process, including myoblast determination protein (MyoD) and myogenin (MyoG) [27]. Thus, we determined whether the LRRK2^G2019S^ mutation initiates muscle regeneration programs. A significantly increased number of PAX7‐positive cells (Figure 6A) was observed in the skeletal muscles of 14mo‐TG mice (by 106%, *p* < 0.0001) but not in the 3mo‐TG mice (Figure 6A). PAX7 is a transcription factor that maintains the activation of satellite cells during impairment [S13].

Similarly, the MyoD positive cells were not increased in the 3mo‐TG mice versus 3mo‐WT mice, but the 14mo‐TG mice exhibited a 55% higher number of MyoD positive cells (*p* < 0.001; Figure 6B) and 380% higher LRRK2 protein level (*p* < 0.001; Figure 6B) compared with 14mo‐WT mice. Western blot results from gastrocnemius muscles also confirmed that the elevated protein expression levels of PAX7 (by 28%, *p* < 0.05; Figure 6C), MyoD (by 71%, *p* < 0.01; Figure 6C) and MyoG (by 41%, *p* < 0.01; Figure 6C) in the 14mo‐TG mice but not altered in the 3mo‐TG mice. These results imply that muscle regeneration programs are initiated during aging in LRRK2^G2019S^ mice.

### 3.7 Defects of Mitochondrial Morphology and Dynamics in the Skeletal Muscles of LRRK2^G2019S^ Mice

**FIGURE 7:**
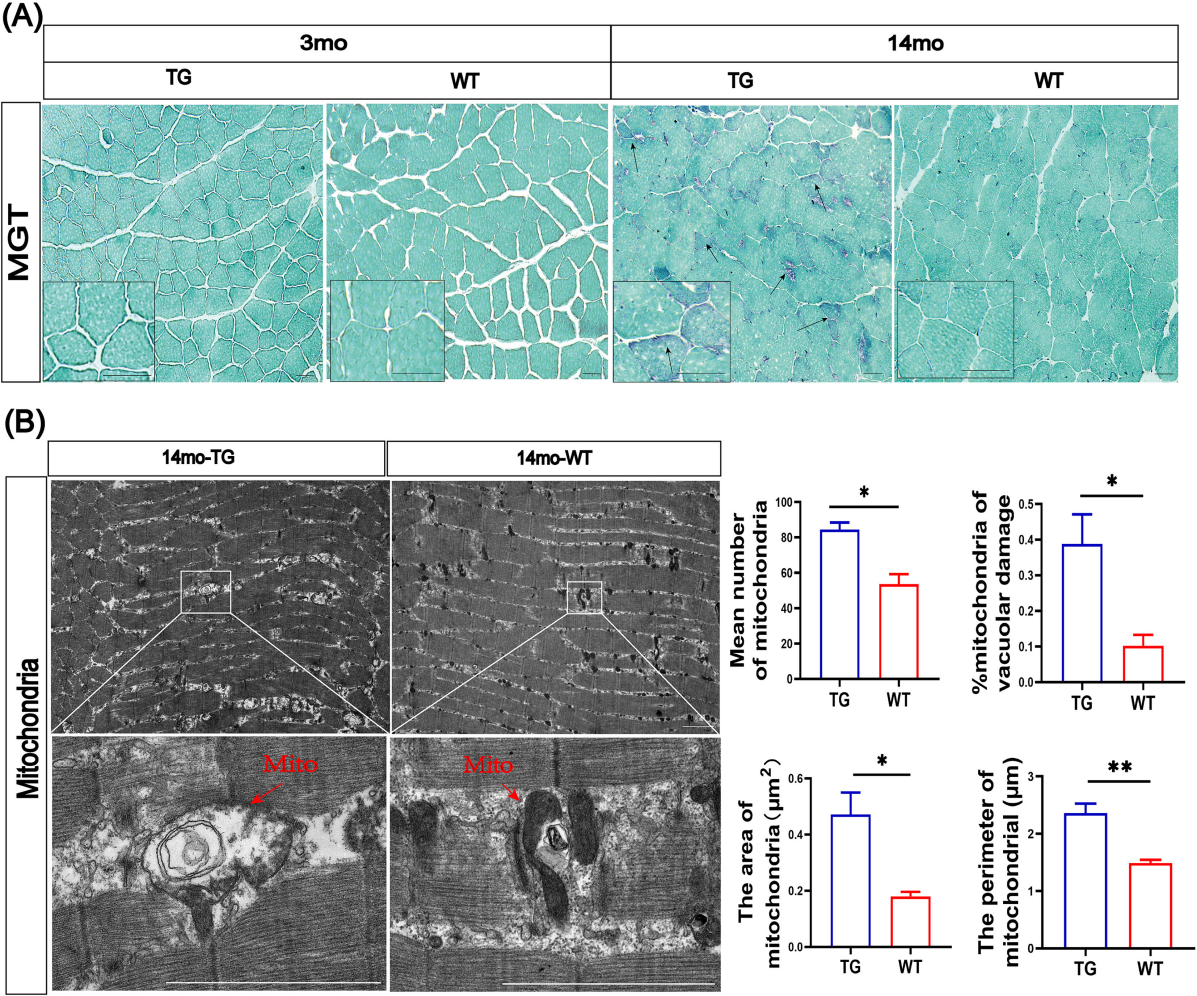
Mitochondrial morphology changes in the skeletal muscles of LRRK2^G2019S^ mice. (A) The representative images of MGT staining of biceps femoris sections show the ragged red fibres (black arrow). Scale bar 50 μm, *n* = 6–8 slices from 3 mice per genotype. (B) The representative TEM images of the mitochondria (red arrow). Scale bar 2 μm, *n* = 10–12 slices per mouse and 3 mice per group. Quantification of the mean number, the proportion of vacuolar damaged, the mean area and mean perimeter of mitochondria. Data were analysed using Student's *t*‐test, presented as the mean ± SEM. ns, non‐significant, *p < 0.05, **p < 0.01.

To determine mitochondrial morphology in the skeletal muscles of TG mice, we assessed the ragged‐red fibres (RRFs) through MGT staining, showing a significant number of abnormal mitochondria in the 14mo‐TG mice, but not in the 3mo‐TG mice (Figure 7A). We further analysed the details of mitochondrial morphology through TEM (Figure 7B) and found that the number of abnormal mitochondria was significantly increased by 58% (*p* < 0.05), showing many swollen mitochondria with damaged cristae (*p* < 0.05; Figure 7B), increased area (up 176%, *p* < 0.05; Figure 7B) and perimeter (up 55%, *p* < 0.01; Figure 7B) in the 14mo‐TG mice as compared with the 14mo‐WT mice.

**FIGURE 8:**
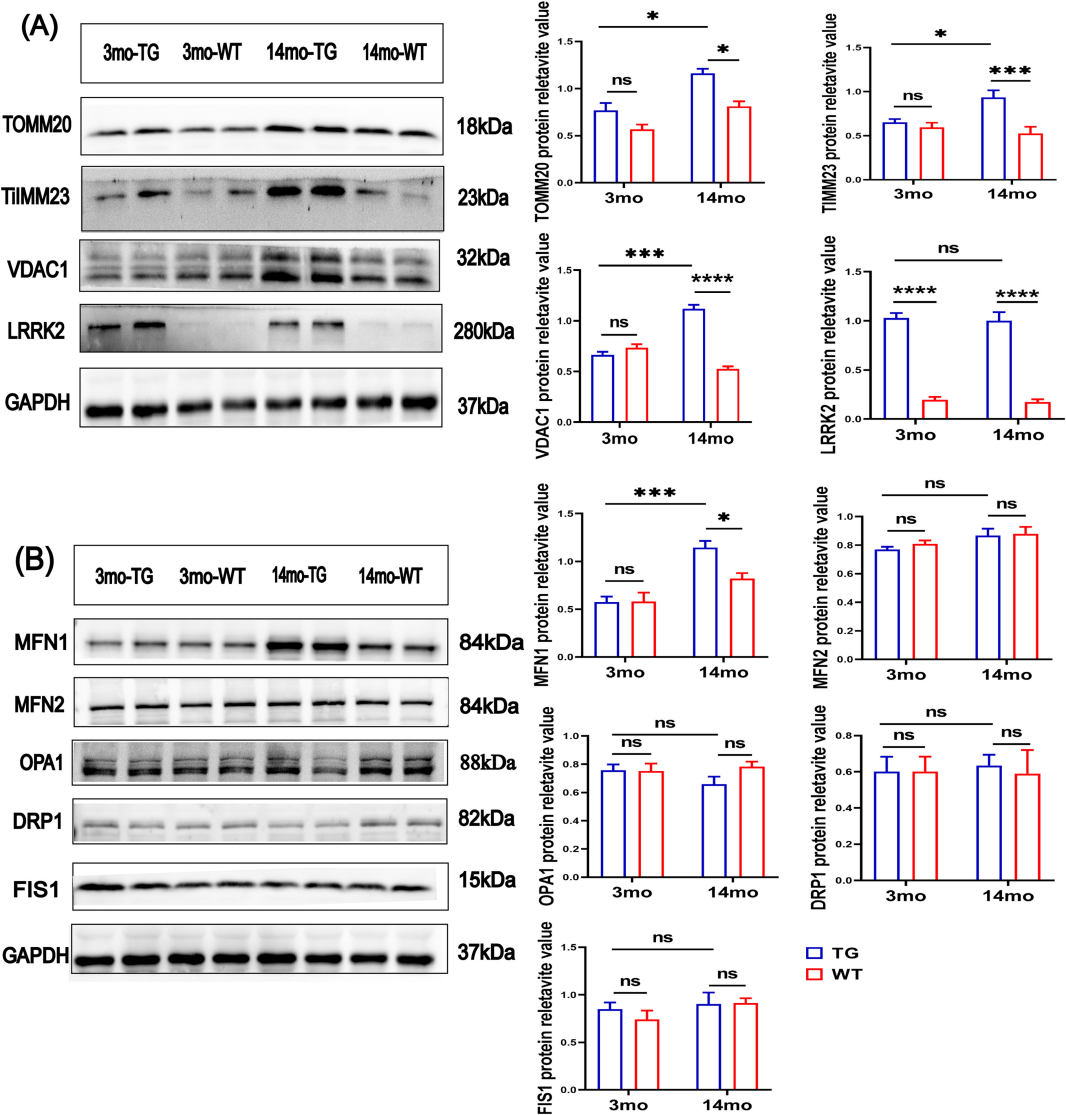
LRRK2^G2019S^ mice display mitochondrial dynamics dysfunction in the skeletal muscles. (A) Western blot analysis for TOMM20, TIMM23, VDAC1, LRRK2 and GAPDH in gastrocnemius muscles, *n* = 4 mice per group. Quantification of TOMM20, TIMM23, VDAC1 and LRRK2 protein expression relative to GAPDH. (B) Western blot analysis for MFN1, MFN2, OPA1, DRP1, FIS1 and GAPDH in gastrocnemius muscles, *n* = 4 mice per group. Quantification of MFN1, MFN2, OPA1, DRP1 and FIS1 protein expression relative to GAPDH. Experiments were repeated three times. Data were analysed by two‐way ANOVA, presented as the mean ± SEM. ns, non‐significant, *p < 0.05, **p < 0.01, ***p < 0.001.

In addition, western blotting (Figure 8A) showed an elevated level of voltage‐dependent anion channel 1 (VDAC1, increased by 115%, *p* < 0.0001), the translocase of outer mitochondrial membrane 20 (TOMM20, increased by 45%, *p* < 0.05) and the translocase of inner mitochondrial membrane 23 (TIMM23, increased by 80%, *p* < 0.001) in the 14mo‐TG mice versus 14mo‐WT mice. However, there was no difference in the 3mo‐TG mice versus 3mo‐WT mice (Figure 8A). These results indicate that mitochondria are impaired in the skeletal muscles of LRRK2^G2019S^ mice. Because mitochondria are dynamic organelles that undergo continuous fission and fusion processes, we analysed the mitochondrial dynamics by measuring the protein levels of fusion proteins mitofusin‐1 (MFN1), fusion proteins mitofusin‐1 (MFN2), optic atrophy 1 (OPA1), fission protein dynamin‐related protein 1 (DRP1) and fission 1 (FIS1) (Figure 8B). The level of MFN1 was significantly increased by 43% (*p* < 0.05; Figure 8B) in the14mo‐TG mice but not in the 3mo‐TG mice compared with age‐matched controls, while the levels of MFN2, OPA1, DRP1 and FIS1 were not altered in both the 3mo‐TG mice and the 14mo‐TG mice (Figure 8B).

## 4 Discussion

Our results first demonstrate that the LRRK2^G2019S^ mice exhibit skeletal muscle impairment accompanied by inflammatory infiltration, mitochondrial morphology changes and dynamic dysfunction during aging. However, myofibre necrosis and inflammatory infiltration may occur at the early stage of the LRRK2^G2019S^ mice. Furthermore, we document the motor behaviour impairment, reduction of gastrocnemius mass and skeletal muscle strength, along with the satellite cells presence at the late stage. In addition, EMG shows muscle membrane impairment and a diminished number of electrically responsive muscle fibres in aged LRRK2^G2019S^ mice. The H&E staining confirms a remarkable myofibre necrosis in the muscle tissues of LRRK2^G2019S^ mice. Furthermore, we observe a reduction in MyHC‐II protein, one of the subtypes of MyHC expressed in the fast myofibres, supporting the possibility of myonecrosis [28]. Moreover, we detect significantly high levels of COX‐2, IL‐1β and CD8a, CD68 positive cells in myofibre, indicating the presence of an abnormal inflammatory process. These results provide new insights into skeletal muscle impairment in the LRRK2^G2019S^‐associated PD and reveal the possible pathophysiological mechanisms.

Notably, aging is an independent risk factor for muscle impairment in LRRK2^G2019S^ mice. During aging, the EMG in 14mo‐TG mice shows increased spontaneous potentials including CRDs and reduced duration of MUAPs, which may reflect an unstable myolemma state [26, S14]. This is similar to the myopathic process but different from typical acute polymyositis or myopathy because of the absence of early recruitment patterns [29]. The TEM analysis shows muscular ultrastructural degeneration, indicating the length of sarcomeres reduction.

Thus, the genetic burden of LRRK2^G2019S^ is age‐dependent not only in the midbrain DAergic neurons [22] but also in the skeletal muscles, though the mechanism remains to be elucidated. The decline in skeletal muscle mass and function is often defined as sarcopenia, an age‐related systemic and progressive disorder with critical pathological processes such as inflammation and mitochondrial integrity [5]. However, the diagnostic criteria for sarcopenia in PD animal models are lacking [30].

Neuroinflammation and immune dysfunction are research hotspots for the pathophysiological mechanisms in PD [29]. The first evidence linking inflammation to PD was the reactive microglia found in the substantia nigra of the post‐mortem tissue from PD patients in 1988 [S15]. Subsequently, an animal model first confirmed the immune‐mediated degeneration of DAergic neurons in guinea pigs [31]. Furthermore, data from the microglia, monocytes, cytokines and memory T cells associated with innate and adaptive immunity may cause immune system dysfunction and dysregulation of different signalling pathways in the pathogenesis of PD [S16, S17]. Increasing evidence suggests that LRRK2 regulates the function of the inflammatory signalling pathway in PD. LRRK2^G2019S^ carriers exhibit higher inflammatory cytokine in their serum and cerebrospinal fluid [32, S18]. The cell cultures and mice studies testified that the LRRK2^G2019S^ is able to up‐regulate the NF‐κB pathway depending on its kinase activity [S19]. Interestingly, our study demonstrates that the pro‐inflammatory cytokines and immune cells in the skeletal muscles are significantly high in the TG mice, suggesting the LRRK2^G2019S^‐associated PD is consistent with peripheral inflammatory pathogenesis.

It is known that myofibre necrosis, macrophage invasion and a sequential inflammatory response can trigger muscle repair and regeneration [33]. After the activation of satellite cells, MyoD is activated for differentiation [S20], and MyoG induces the formation of new myotubes for terminal differentiation [S21] during impairment. The increased expression of MyoD and LRRK2 protein in our old TG mice may indicate that LRRK2 protein participates in the impairment and repair process. Thus, our results emphasize that LRRK2^G2019S^ participates in myofibre regeneration in a chronic process, which is similar to chronic skeletal muscle diseases such as Duchenne muscular dystrophy, showing the satellite cells continue increasing [S22].

Proverbially, the muscle function is highly reliant on mitochondrial metabolism [S23]. Therefore, we found profound RRFs, damaged mitochondria and expression levels of TOMM20 and TIMM23 protein in skeletal muscles in LRRK2^G2019S^ mice. Mitochondria are double‐membraned organelles that constantly undergo homeostatic processes of fission and fusion [34, S24]. These processes are influenced by aging, genetic background and cellular homeostasis [S25]. Mitochondrial fusion requires dynamin‐like GTPase proteins MFN1, MFN2 and OPA1, bringing mitochondrial elongation. Fission fragments are mediated by DRP1 and several other proteins, such as FIS1 [S26]. Recent studies have shown that LRRK2^G2019S^ is located in the kinase domain and enhances the kinase activity [35], mediating protein–protein interactions. It partially co‐localizes with MFN1 and binds it to the GTPase domain in LRRK2 protein [36], regulating mitochondrial membrane fusion and fission events. In our 14mo‐TG mice, the expression level of MFN1 was markedly increased. Overexpression of MFN1 can cause the formation of large mitochondria [S27]. Several studies have described the swelling mitochondria as the abnormal morphology in pathological conditions with the enriched MFN1 expression [S28]. Punch skin biopsies from PD patients with LRRK2^G2019S^ mutation display mitochondria elongated [S29]. LRRK2 knockout macrophages increase mitochondrial stresses and fragmentation [S30]. Similarly, LRRK2^G2019^ knockin mice demonstrate mitochondrial abnormalities with fission arrest in the striatum [S31]. Furthermore, swelling or large mitochondria cannot be cleared by mitophagy, leading to caspase‐dependent cell death [S32].

Interestingly, our data show that the expression level of VDAC1 significantly increased in the 14mo‐TG mice. VDAC1 is a multifunctional channel protein that acts as the mitochondrial gatekeeper, is located in the outer mitochondrial membrane (OMM), and controls cell metabolism, energy and signalling pathways in mitochondria [37]. As the sole channel mediating metabolites through the OMM to the cytosol, including over 100 proteins, nucleotides, fatty acids, cholesterol, ions and other metabolites [S33]. Several reports have confirmed that overexpression of VDAC1 triggers cell death [38, S34], including myocytes [39, S35]. Here, we demonstrate that LRRK2^G2019S^ may enhance the expression of MFN1 to promote fusion during aging, which can cause the swelling of mitochondria into a pathological state. Besides, overexpression in VDAC1 might switch the metabolism and promote necrosis in the skeletal muscle cells.

This study has some limitations. Firstly, the animal model applied in this study is the LRRK2^G2019S^ transgenic mice, as the mutations in this gene can cause PD in humans. It is unknown if other genetic defects‐caused PD or sporadic PD have similar muscular pathology. Further studies are needed to investigate other PD‐causing genetic defects in animal models and PD patients. Secondly, little is clear about the underlying mechanisms of the LRRK2^G2019S^ gene mutation that causes muscular pathology. More studies are required to uncover the mechanisms in this regard. Additionally, aging is an independent risk factor. In future studies, we need to explore how aging factors influence the pathogenic effect of LRRK2^G2019S^ gene mutation on muscles. Moreover, EMG examination and genetic counselling might be useful in PD patients presenting with complaints of fatigue, muscular pain, weakness and even dysphagia. Generally, a cohort study of patients with LRRK2^G2019S^ mutation is warranted to confirm our findings.

## 5 Conclusion

Skeletal muscle impairment in PD may cause patients suffering from severe disability and high mortality. We demonstrate that the PD mouse model carrying LRRK2^G2019S^ mutation displays myofibre necrosis and regeneration, inflammatory infiltration, and muscular mitochondrial morphology changes and dynamics dysfunction during aging. We are the first to characterize the EMG profile in LRRK2^G2019S^ mice with skeletal muscle changes. Our results may expand the view that PD is a systemic disease involving not only the central nervous system but also peripheral organs, such as skeletal muscles. Taken together, our LRRK2^G2019S^ mice may serve as a valuable model for the pathology and possible pathogenesis of skeletal muscle impairment in PD.

## Ethics Statement

Animal care was reviewed and approved by Laboratory Animal Care Guidelines approved by the Institutional Animal Care Committee at Dalian Medical University. The protocol was approved by the Institutional Animal Care Committee at Dalian Medical University. The authors of this manuscript certify that they comply with the ethical guidelines for authorship and publishing in the *Journal of Cachexia, Sarcopenia and Muscle* [40].

## Conflicts of Interest Statement

The authors declare no conflicts of interest.

## Supporting information


**Data S1** Supporting Information.

**Data S2** Supporting Information.
