# Supplementary material for: LRRK2G2019S Gene Mutation Causes Skeletal Muscle Impairment in Animal Model of Parkinson's Disease
Source: J Cachexia Sarcopenia Muscle. 2024 Sep 23;15(6):2595–607. doi: 10.1002/jcsm.13604 (PMC11634472; doi:10.1002/jcsm.13604)
Supplement: Supplementary file 1 — Data S1 Supporting Information. [file JCSM-15-2595-s002.docx]

**Supplementary materials and methods**

**Animals**

The TG mice and their littermate wild-type (WT) mice of 3 and 14-month-old were used in this study, and male TG mice were hybridized with female WT mice to produce offspring. The genotype of mice was identified by polymerase chain reaction (PCR) assay through tail biopsies at 21 days. The sequence of the transgene forward primer is 5' GAC TAC AAA GAC GAT GAC GAC AAG 3' and the transgene reverse primer is 5' CTA CCA CCA CCC AGA TAA TGTC 3'. The sequence of internal control forward primer is 5' CAA ATG TTG CTT GTC TGG TG 3' and the internal control reverse primer is 5' GTC AGT CGA GTG CAC AGT TT 3'. The PCR program is 37×(94℃30s, 53℃30s, 72℃30s).

**Behavioral analysis**

All four groups of mice (3mo-TG, 3mo-WT, 14mo-TG and 14mo-WT) were examined by rotarod test, pole-climbing test, and forelimb grip strength test. Animals were adapted to the testing room environment for 1 hour before test.

Rotarod test was taken to assess the motor balance as described previously^1^. The mice were trained on an accelerating rotarod (IITC Life Science, Woodland Hills, USA) between 9:00 and 14:00 for five consecutive days, three times daily, with 1 hour rest interval between sessions. During training, the speed started at 4 revolutions per minute (rpm) and accelerated to 40 rpm at 10 rpm. The testing was conducted on the sixth day, and the time on the road before falling was recorded for analysis.

Next, a pole-climbing test was performed to measure motor coordination and balance dominated by forelimb strength in a quiet room. A 3 cm diameter rubber ball was fixed on the top of a wooden rod with a length of 60 cm and a diameter of 1cm. The wooden rod was wrapped with gauze to prevent slipping. Each mouse was placed on the ball with its head upward, allowed to turn around and climb to the ground. Three trials per day with at least 1 hour of rest for five consecutive days, recorded the time spent turning around and climbing down from the top to the ground as the total time for analysis on the sixth day.

The forelimb grip strength test was used to measure the strength of the forelimbs^2^. Before the test, each mouse was weighted for standardization. A grid was connected to a force transducer, grabbed the mouse tail gently and ensure both forelimbs grasped the grid tightly. Pulled the mouse horizontally away from the grid, and the transducer notes the force. The pull was repeated three times with a one-minute resting period between sessions^3^. Calculated the average values of the three measurements, standardized by body weight, and expressed the results as N/g (Newtons/ grams Body weight) for analysis.

**Electromyography**

EMG recordings provide an objective tool for differential localization diagnosis on neuromuscular disease and evaluate the disease course^4^. In our study, we used nEMG, motor nerve conduction velocity (MCV), and repetitive nerve stimulation (RNS) to examine the electroactivity in gastrocnemius muscle and sciatic nerve for all four groups’ mice (3mo-TG, 3mo-WT, 14mo-TG and 14mo-WT). All data were recorded and collected with a multi-channel EMG system (Medelec Synergy, Natu's Neurology, United States).

First, we applied oxybuprocain hydrochloride on the skin of the left gastrocnemius muscle for surface aesthesia over 15 minutes. Based on the previous study^5,6^, the left lower limb was gently pulled to collect more than 12 motor unit action potentials (MUAPs) per mouse, then we analyzed the average duration and the number of phases. Clamping the tail instantly to obtain the recruitment potentials as the gastrocnemius muscle was contracting tensely, and the amplitudes were noted for analysis. Immediately, we anesthetized the mice with isoflurane (R500ie, RWD, China) inhalation and recorded spontaneous discharges, including fibrillation potentials, positive sharp waves, and complex repetitive discharges (CRDs). Data were collected from at least four directions every muscle for analysis.

Besides, we measured the MCV of sciatic nerve as previously described^6^, recorded the conduction velocity (CV), the amplitude and the latency of compound muscle action potentials (CMAPs). RNS data were obtained after the MCV test, recording the decreased percent by the fifth CMAP to the first in low-frequency (3Hz 10 times,5Hz 10 times) and the increased percent by the last CMAP to the first in high-frequency^4^ per mouse.

**Histological examination**

We conducted general histological examination for all four groups’ mice (3mo-TG, 3mo-WT, 14mo-TG and 14mo-WT). After anesthesia with isoflurane, mice were perfused with a pre-cooled phosphate buffer solution (PBS, 0.1M, pH 7.2). The gastrocnemius muscle (excluding the side used for EMG examination) and biceps femoris muscles were quickly isolated, weighted and then frozen in isopentane with liquid nitrogen for 20s^7^. Removed the muscle from the isopentane, embedded by optimal cutting temperature compound, and cut into 10 μm thick sections in the cryostat (CM-1950S, Leica, Germany). For pathological changes, sections from biceps femoris muscles were stained with hematoxylin and eosin staining (H＆E, C0105S, Beyotime, China) and modified gomori trichrome (MGT, G3510, Solarbio, China) according to our previously described^8^. The sections were photographed by light/fluorescent microscopy (Ix81, Olympus, Japan) under 40×objective lenses. Image J software (Rockville, United States) was used to measure the average myofiber size, the number of inflammation and necrosis myofibers in H＆E staining.

**Immunohistochemical Staining**

For further investigation of the inflammatory infiltration pathology, the anti-CD4, anti-CD8a, anti-CD68 (Supplementary Table 1) were used for immunohistochemical (IHC) staining as described previously^9^. The frozen 10 μm thick sections obtained from the biceps femoris muscles of four groups’ mice (3mo-TG, 3mo-WT, 14mo-TG and 14mo-WT). The sections were first fixed with 4% pre-cooled paraformaldehyde (PFA) for more than 10min and washed with PBS (5 min×3 time). Then, incubated with 1% H_2_O_2_ for 10 minutes and washed with PBS, incubated with anti-CD4, anti-CD8a, anti-CD68 overnight at 4℃. After extensive washed, incubated with goat anti-rat IgG (Pv-9004, Zsbio, China), goat anti-rabbit IgG (Pv-9001, Zsbio, China) for 1 hour at room temperature. Washed with PBS and visualized with diaminobenzidine reagent (ZLI-9019, Zsbio, China) for 8min, washed with distilled water, dehydrated, cleared, and mounted. Images were captured with light/fluorescent microscopy (Ix81, Olympus, Japan) under 40 ×and 60× objective lenses; image J software (Rockville, United States) was used to calculate the positive stained cells.

**Immunofluorescence Staining**

We further accessed the immunofluorescence (IF) staining investigation for the pathology of type II muscle fiber changes and regeneration. The frozen 10 μm thick sections obtained from biceps femoris muscles of four groups’ mice (3mo-TG, 3mo-WT, 14mo-TG and 14mo-WT) were fixed with 4% pre-cooled PFA for more than 10 minutes, washed with PBS three times and incubated with blocking buffer (5% normal goat serum, 0.2% Triton-X 100, and 0.05% NaN3 in PBS) for 1 hours at room temperature. Then, incubated with the primary antibodies: anti-MYHC-II, anti-Lamininβ_1_, anti-Pax7, anti-Myod, anti-LRRK2 (Supplementary Table1) overnight at 4 °C. The sections were washed thoroughly with PBS and incubated with secondary antibodies: Alexa Fluor-conjugated goat anti-rabbit (1:2000, 8889S/4412S, CST, United States), Alexa Fluor-conjugated goat anti-mouse (1:2000, 4408S/8890S, CST, United States) for 1 hour at room temperature avoid light. Then sections were mounted by antifade mounting medium with DAPI (P0126, Beyotime, China). Finally, the sections were imaged using a laser scanning confocal microscope (A1confocal, Nikon, Japan) under 60× objective lenses. Image J software (Rockville, United States) was used to calculate the mean density fluorescence.

**Transmission electron microscopy analysis**

Two groups’ mice (14mo-TG and 14mo-WT) in our study were examined by transmission electron microscopy (TEM). After the mouse was executed in 1-3 minutes, gastrocnemius muscle tissue (1mm^3^) was taken quickly into a transmission electron microscopy (TEM) fixative solution (G1102, Service, China). Then, washed the tissues with PBS (0.1M PH7.4) for 15 min×3 times, the muscle tissues avoid light and post-fixed with 1% osmium acid for 2 hours at room temperature. After a graded series of dehydrated, embedding steps^10^, the blocks were cut into 60-80nm thick in the ultramicrotome (Leica UC7, Leica, Germany) and stained with 2% uranium acetate and 2.6% lead citrate; the stained slices were observed in each mouse under TEM (Ht7700, Hitachi, Japan). The sarcomere, A band, I band, H zone, Z disk, M band, the area of mitochondria, and the number of vacuolar mitochondria were analyzed by using Image J software (Rockville, United States).

**Protein extraction and western blotting**

We performed western blotting in the gastrocnemius muscle extracts obtained from four groups’ mice (3mo-TG, 3mo-WT, 14mo-TG and 14mo-WT) to determine the inflammation and mitochondrial function at the molecular levels. According to our previously reported described^7^, total protein was extracted from 20mg fresh gastrocnemius muscle mixed with 20μl pre-cooled RIPA lysis buffer (P0126, Beyotime, China), containing 1% protease inhibitor cocktails (P8340, Sigma-Aldrich, United States), homogenized thoroughly by a cryogenic grinder (Jxfstprp-II-02, Jingxin, China). Then, samples were lysed on ice for 30 min, centrifuged (12000g, 15min, 4℃), collected the supernatant for BCA protein concentration measurement (T9300A, Takara, China), proteins were mixed with the loading buffer (LT101, Epizyme, China) and boiled at 95℃ for 10 minutes.

The equal quality of proteins was loaded on 10% or 12.5% sodium dodecyl sulfate-polyacrylamide gel electrophoresis (SDS-PAGE) and transferred to a polyvinylidene fluoride (PVDF) membrane (Millipore, Bedford, United States). After blocking with 5% fat-free milk for 2 hours at room temperature, we washed the PVDF membrane in Tris-buffered saline-Tween (TBST) three times, and then incubated with the primary antibodies (Supplementary Table1) overnight at 4°C. Washed with TBST thoroughly before incubating with secondary antibody: goat anti-mouse HRP (RGAM001, Protentech, China), goat anti-rabbit HRP (RGAR001, Protentech, China) for 1 hour at room temperature. Finally, the membrane was incubated with enhanced chemiluminescence (Superkine™, Abbkine, China), and the protein bands were quantified using the chemiluminescence system (Hongtao, Beijing, China).

**References:**

1. Liu X, Yu H, Wang Y, et al. Altered Motor Performance, Sleep EEG, and Parkinson’s Disease Pathology Induced by Chronic Sleep Deprivation in Lrrk2G2019S Mice. *Neurosci. Bull.* 2022; 38:1170-1182.

2. Maaike VP, Annemieke A-R. Assessing Functional Performance in the Mouse Model. *J Vis Exp* 2014:e51303.

3. Zhang S, Yan H, Ding J, et al. Skeletal muscle‐specific DJ‐1 ablation‐induced atrogenes expression and mitochondrial dysfunction contributing to muscular atrophy. *J Cachexia Sarcopenia Muscle* 2023; 14:2126-2142.

4. Stålberg E, van Dijk H, Falck B, et al. Standards for quantification of EMG and neurography. *Clin. Neurophysiol.* 2019; 130:1688-1729.

5. Han JJ, Carter GT, Ra JJ, Abresch RT, Chamberlain JS, Robinson LR. Electromyographic studies in mdx and wild‐type C57 mice. *Muscle Nerve* 2005; 33:208-214.

6. Van Den Bosch L, Van Damme P, Robberecht W, Prior R, Pollari E. Electrophysiological Measurement of Compound Muscle Action Potential from the Forelimbs in Mouse Models of Motor Neuron Degeneration. *J Vis Exp* 2018:e57741.

7. Jing ZJ, Ming ZQ, Sheng C, Dong LW. Repurposing carbamazepine for the treatment of amyotrophic lateral sclerosis in SOD1‐G93A mouse model. *CNS Neurosci. Ther.* 2018; 24:1163-1174.

8. Song L, Gao Y, Zhang X, Le W. Galactooligosaccharide improves the animal survival and alleviates motor neuron death in SOD1G93A mouse model of amyotrophic lateral sclerosis. *Neuroscience* 2013; 246:281-290.

9. Wang Y, Chen X, Wang Y, Li S, Cai H, Le W. The essential role of transcription factor Pitx3 in preventing mesodiencephalic dopaminergic neurodegeneration and maintaining neuronal subtype identities during aging. *Cell Death Dis.* 2021; 12:1008-1019.

10. Wang P, Chen X, Wang Y, et al. Essential role for autophagy protein VMP1 in maintaining neuronal homeostasis and preventing axonal degeneration. *Cell Death Dis.* 2021; 12:116-120.

**Table 1 Antibodies used in this study**

| Target | Application | Dilution | Company | Cat. Number |
| --- | --- | --- | --- | --- |
| MYHC2 | IF | 1:200 | Santa Cruz | sc-53095 |
| Lamininβ_1_ | IF | 1:200 | Proteintech | 23498-1- ap |
| Pax7 | IF | 1:200 | Proteintech | 20570-1- ap |
| Pax7 | WB | 1:1000 | Proteintech | 20570-1- ap |
| Myod | IF | 1:200 | Santa Cruz | sc-377460 |
| Myod | WB | 1:1000 | Santa Cruz | sc-377460 |
| LRRK2 | IF | 1:100 | Abcam | ab133474 |
| LRRK2 | WB | 1:1000 | Abcam | ab133474 |
| CD4 | IHC | 1:200 | Thermofisher | 14-0041-81 |
| CD8a | IHC | 1:200 | Thermofisher | 13-0081-81 |
| CD68 | IHC | 1:200 | Service | gb113109100 |
| IL-6 | WB | 1:1000 | Santa Cruz | sc-57315 |
| IL-1β | WB | 1:1000 | SigmaAldrich | I3767-1mg |
| TNF-α | WB | 1:1000 | Proteintech | 17590-1-ap |
| Cox2 | WB | 1:1000 | Santa Cruz | sc-19999 |
| Myogenin | WB | 1:1000 | Santa cruze | sc-12732 |
| Tomm20 | WB | 1:1000 | Abcam | ab136785 |
| Timm23 | WB | 1:1000 | Santa cruze | sc-514433 |
| Mfn1 | WB | 1:1000 | Proteintech | 13798-1-ap |
| Mfn2 | WB | 1:1000 | Santa cruze | sc-515647 |
| FIS1 | WB | 1:1000 | Cell signaling | 32525 |
| Drp1 | WB | 1:5000 | Proteintech | 12957-1-ap |
| OPA1 | WB | 1:2000 | Cell signaling | 80471 |
| VDAC1 | WB | 1:2000 | Cell signaling | 4661 |
| GAPDH | WB | 1:4000 | Cell signaling | 2118 |
