# Supplementary material for: LRRK2G2019S Gene Mutation Causes Skeletal Muscle Impairment in Animal Model of Parkinson's Disease
Source: J Cachexia Sarcopenia Muscle. 2024 Sep 23;15(6):2595–607. doi: 10.1002/jcsm.13604 (PMC11634472; doi:10.1002/jcsm.13604)
Supplement: Supplementary file 2 — Data S2 Supporting Information. [file JCSM-15-2595-s001.docx]

**References**

S1. Whittaker K, Schroeter N, Rijntjes M, et al. Severe camptocormia due to myositis of paraspinal muscles as an early manifestation of Parkinson's disease. *Parkinsonism Relat. Disord.* 2018; 46:95-97.

S2. Hemmi S, Kurokawa K, Izawa N, Kutoku Y, Murakami T, Sunada Y. Dramatic Response of Dropped Head Sign to Treatment with Steroid in Parkinson's Disease: Report of Three Cases. *Intern. Med.* 2011; 50:757-761.

S3. Gdynia H-J, Sperfeld A-D, Unrath A, et al. Histopathological analysis of skeletal muscle in patients with Parkinson's disease and ‘dropped head’/‘bent spine’ syndrome. *Parkinsonism Relat. Disord.* 2009; 15:633-639.

S4. Wrede A, Margraf NG, Goebel HH, Deuschl G, Schulz-Schaeffer WJ. Myofibrillar disorganization characterizes myopathy of camptocormia in Parkinson’s disease. *Acta Neuropathol.* 2011; 123:419-432.

S5. Starrfelt R, Baille G, Perez T, et al. Early occurrence of inspiratory muscle weakness in Parkinson’s disease. *PLoS One* 2018; 13:e0190400.

S6. Blin O, Desnuelle C, Rascol O, Borg M, Paul HPS, Azulay jp. Mitochondrial respiratory failure in skeletal muscle from patients with Parkinson's disease and multiple system atrophy. *J. Neurol. Sci.* 1994; 125:95-101.

S7. Lavin KM, Sealfon SC, McDonald M-LN, et al. Skeletal muscle transcriptional networks linked to type I myofiber grouping in Parkinson’s disease. *J. Appl. Physiol.* 2020; 128:229-240.

S8. Tolosa E, Vila M, Klein C, Rascol O. LRRK2 in Parkinson disease: challenges of clinical trials. *Nat. Rev. Neurol.* 2020; 16:97-107.

S9. Wallings RL, Tansey MG. LRRK2 regulation of immune-pathways and inflammatory disease. *Biochem. Soc. Trans.* 2019; 47:1581-1595.

S10. Civiero L, Cogo S, Biosa A, Greggio E. The role of LRRK2 in cytoskeletal dynamics. *Biochem. Soc. Trans.* 2018; 46:1653-1663.

S11. Tansey MG, Wallings RL, Houser MC, Herrick MK, Keating CE, Joers V. Inflammation and immune dysfunction in Parkinson disease. *Nat. Rev. Immunol.* 2022; 22:657-673.

S12. Cabezudo D, Tsafaras G, Van Acker E, Van den Haute C, Baekelandt V. Mutant LRRK2 exacerbates immune response and neurodegeneration in a chronic model of experimental colitis. *Acta Neuropathol.* 2023; 146:245-261.

S13. Nogueira JM, Hawrot K, Sharpe C, et al. The emergence of Pax7-expressing muscle stem cells during vertebrate head muscle development. *Front. Aging Neurosci.* 2015; 7:62-84.

S14. Lesley K, Bethany J, Colin H. Clinical significance of complex repetitive discharges: a case-control study. *Muscle Nerve* 2003; 28:504-507.

S15. McGeer P, Itagaki S, BE. Boyes M, McGeer B. Reactive microglia are positive for HLA-DR in the substantia nigra of Parkinson's and Alzheimer’s disease brains. *Neurology* 1988; 38:1285-1291.

S16. Tan E, Chao Y, West A, Chan L, Poewe W, Jankovic J. Parkinson disease and the immune system — associations, mechanisms and therapeutics. *Nat. Rev. Neurol.* 2020; 16:303-318.

S17. Brochard V, Combadière B, Prigent A, et al. Infiltration of CD4+ lymphocytes into the brain contributes to neurodegeneration in a mouse model of Parkinson disease. *J. Clin. Invest.* 2009; 119:182-192.

S18. Lopez de Maturana R, Aguila JC, Sousa A, et al. Leucine-rich repeat kinase 2 modulates cyclooxygenase 2 and the inflammatory response in idiopathic and genetic Parkinson's disease. *Neurobiol. Aging* 2014; 35:1116-1124.

S19. López de Maturana R, Lang V, Zubiarrain A, et al. Mutations in LRRK2 impair NF-κB pathway in iPSC-derived neurons. *J. Neuroinflammation* 2016; 13:295-310.

S20. Asfour HA, Allouh MZ, Said RS. Myogenic regulatory factors: The orchestrators of myogenesis after 30 years of discovery. *Exp. Biol. Med.* 2018; 243:118-128.

S21. Fernández-Lázaro D, Garrosa E, Seco-Calvo J, Garrosa M. Potential Satellite Cell-Linked Biomarkers in Aging Skeletal Muscle Tissue: Proteomics and Proteogenomics to Monitor Sarcopenia. *Proteomes* 2022; 10:29-47.

S22. Bockhold KJ, Rosenblatt JD, Partridge TA. Aging normal and dystrophic mouse muscle: Analysis of myogenicity in cultures of living single fibers. *Muscle Nerve* 1998; 21:173–183.

S23. Quadrilatero J. Mitochondria: Key modulators of skeletal muscle remodeling. *Semin. Cell Dev. Biol.* 2023; 143:1-2.

S24. Hong X, Isern J, Campanario S, et al. Mitochondrial dynamics maintain muscle stem cell regenerative competence throughout adult life by regulating metabolism and mitophagy. *Cell Stem Cell* 2022; 29:1298-1314.

S25. Chen H, Vermulst M, Wang YE, et al. Mitochondrial Fusion Is Required for mtDNA Stability in Skeletal Muscle and Tolerance of mtDNA Mutations. *Cell* 2010; 141:280-289.

S26. Adebayo M, Singh S, Singh AP, Dasgupta S. Mitochondrial fusion and fission: The fine‐tune balance for cellular homeostasis. *FASEB J.* 2021; 35:e21620.

S27. López Doménech G, Howden JH, Covill Cooke C, et al. Loss of neuronal Miro1 disrupts mitophagy and induces hyperactivation of the integrated stress response. *EMBO J.* 2021; 40:e100715.

S28. Mortiboys H, Johansen KK, Aasly JO, Bandmann O. Mitochondrial impairment in patients with Parkinson disease with the G2019S mutation in LRRK2. *Neurology* 2010; 75:2017-2020.

S29. Weindel CG, Bell SL, Vail KJ, West KO, Patrick KL, Watson RO. LRRK2 maintains mitochondrial homeostasis and regulates innate immune responses to Mycobacterium tuberculosis. *eLife* 2020; 9:e51071.

S30. Yue M, Hinkle KM, Davies P, et al. Progressive dopaminergic alterations and mitochondrial abnormalities in LRRK2 G2019S knock-in mice. *Neurobiol. Dis.* 2015; 78:172-195.

S31. Sun K, Jing X, Guo J, Yao X, Guo F. Mitophagy in degenerative joint diseases. *Autophagy* 2020; 17:2082-2092.

S32. Shoshan Barmatz V, Pittala S, Mizrachi D. VDAC1 and the TSPO: Expression, Interactions, and Associated Functions in Health and Disease States. *Int. J. Mol. Sci.* 2019; 20:3348-3355.

S33. Shoshan-Barmatz V, De Pinto V, Zweckstetter M, Raviv Z, Keinan N, Arbel N. VDAC, a multi-functional mitochondrial protein regulating cell life and death. *Mol. Aspects Med.* 2010; 31:227-285.

S34. Shoshan Barmatz V, Krelin Y, Shteinfer Kuzmine A. VDAC1 functions in Ca2+ homeostasis and cell life and death in health and disease. *Cell Calcium* 2018; 69:81-100.

S35. Flores-Romero H, Dadsena S, García-Sáez AJ. Mitochondrial pores at the crossroad between cell death and inflammatory signaling. *Mol. Cell* 2023; 83:843-856.
